# Pattern of physical exercise practice among university students in the Kingdom of Saudi Arabia (before beginning and during college): a cross-sectional study

**DOI:** 10.1186/s12889-019-8093-2

**Published:** 2019-12-21

**Authors:** Sarah Ahmed Alkhateeb, Najwa Fahad Alkhameesi, Ghadeer Nazeh Lamfon, Shahad Zuhair Khawandanh, Lujain Khalid Kurdi, Murooj Yaseen Faran, Alya Abdullah Khoja, Lujain Muhammed Bukhari, Hadeel Rajeh Aljahdali, Nuha Abdullah Ashour, Hessan Turki Bagasi, Raghdah Adel Delli, Ohood Abdullah Khoja, Osama Yousof Safdar

**Affiliations:** 1 Faculty of Medicine, King Abdulaziz University, Altahlia, PO Box14071, Jeddah, 21414 Saudi Arabia; 2 Pediatric Nephrology Centre of Excellence, Faculty of Medicine, King Abdulaziz University, Altahlia, PO Box14071, Jeddah, 21414 Saudi Arabia

**Keywords:** Saudi Arabia, Sport, King Abdulaziz University, Awareness

## Abstract

**Background:**

Transition to college is characterized by change, ambiguity, and adjustment compared to the previous lifestyle before entering college. Our study aimed was to determine the pattern of students’ physical exercise practices in the Kingdom of Saudi Arabia in the period before and during college attendance.

**Methods:**

A cross-sectional study was conducted among university students from fifteen universities in the Kingdom of Saudi Arabia on August 2017. The data were collected using an electronic questionnaire that was modified from questionnaires used in previous studies. Statistical analysis and data entry was performed with SPSS version 21. This study was approved by the Research Ethics Committee at King Abdulaziz University.

**Results:**

417 college students completed the questionnaire and 77.2% were female. Slightly more than half of the participants (55.9%) were from King Abdulaziz University, and 59.7% were from healthcare specialties from different institutions. The mean age of the participants was 21.80 years old (*SD* = 2.75). The pattern of practicing physical exercise showed a significant decrease after enrollment in college (*p* = 0.000). The most common reason for not practicing exercise was time restrictions, accounting for 18.5% of all the reasons, while the most common reason for practicing exercise was to improve body shape, accounting for 48% of all the reasons.

**Conclusions:**

Our research found that there was a significant decrease in doing regular exercise during one’s college years in comparison to the school years prior to college. To address the decrease in physical exercise, we recommend organizing and promoting more awareness campaigns and providing suitable sports facilities and infrastructure.

## Background

Graduation from high school and going to college or a university is a major life change that many individuals must face [1]. This transition has been studied as a process, not only as an event, and the process can be different from one individual to the next [1, 2]. Transition to college is characterized by change, ambiguity, and adjustment compared to the previous lifestyle before entering college. Furthermore, students’ identity formation is affected during this transition by considering personal responsibility and making an independent decision [3]. As such, transitioning to college life could be stressful due to an increased pressure to focus on academic performance and having a new social life, as well as in some instances moving away from home for the first time. College students are vulnerable to experiencing tension, stress, and anxiety, and exercise could be used as an excellent method to manage these conditions [4]. A meta-analysis of previous studies regarding physical activity among college students found that approximately 40–50% were inactive [5]. Additionally, in the transition to college, a significant decline in physical activity, in general, has been observed [1, 6], especially in performing physical exercise [4, 7]. Exercise is a subcategory of physical activity that is intentioned, regular, and structured, and the aim is to improve or maintain physical fitness [8]. Moreover, university students are at risk for weight gain 5.5 times more than the general population [7, 9, 10]. They are also at risk of developing high blood pressure and high serum cholesterol [11]. Therefore, exercise is not only helpful in managing psychological stress reactions that students can experience, but it also has a positive effect on mood and self-esteem, physical and mental health, and the student’s quality of life [4, 12]. Furthermore, it was shown that exercise prevents 35 chronic conditions [12]. Two studies were conducted in Saudi Arabia that concerned physical activity generally but not exercise behaviors specifically [13, 14]. Also, one of the studies was interested in women only [14], and neither study addressed practicing sports among university students in Saudi Arabia. Therefore, the present study aimed to determine the pattern of practicing sports and physical exercise among university students in the Kingdom of Saudi Arabia, before and while attending college.

## Methods

**Fig. 1:**
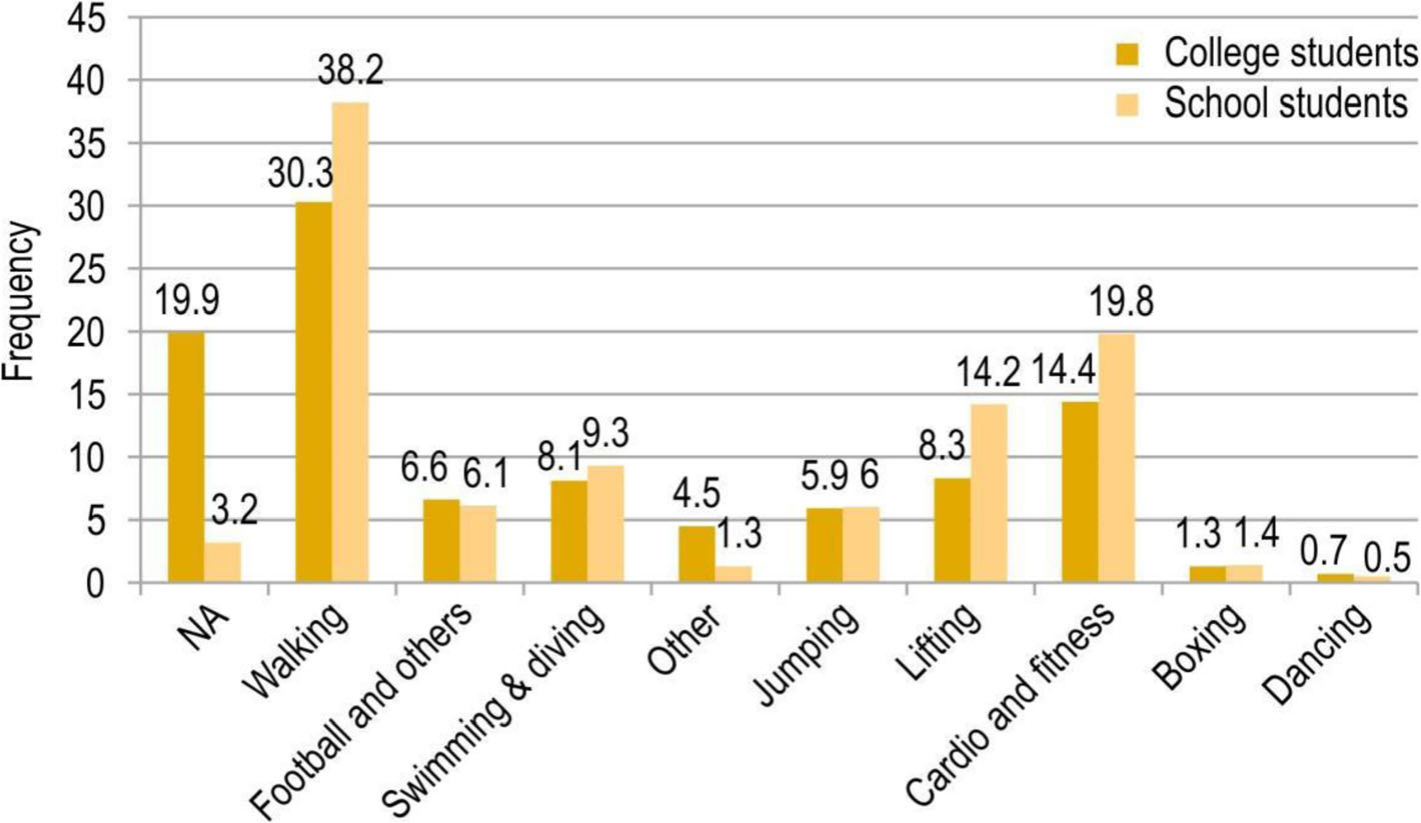
sporting type

A cross-sectional study was conducted among university students in the Kingdom of Saudi Arabia on August 2017. This study was approved by the Research Ethics Committee at King Abdulaziz University. No minors below 16 years were included in the study. University students from 15 universities were invited to participate in the study (including all colleges and academic years) (Fig. 1). Data were collected using an electronic questionnaire that was posted on social media sites (Twitter, Telegram, and WhatsApp groups). The necessary sample size was calculated using Raosoft (http://www.raosoft.com/samplesize.html) with the ideal sample size estimated to be 385 participants, based on a population size of183,784 college students in Saudi Arabia,5% error, a 95% confidence level, and 50% problem prevalence. The research members used items from questionnaires from four previous studies [13–16] and arranged the items to assess the pattern of physical exercise and sports before and during the participants’ college years. For example, Musaiger et al. [16] were used for items on barriers to exercising and sports, El-Gilany et al. [15] was used for items on barriers and benefits, Alsubaie and Omer [13] and Khalaf et al. [14] provided items on reasons and places. The questionnaire consisted of 34 multiple-choice questions divided into three parts. The first part concerned general demographic and health information including age, gender, marital status, university, college, academic year, chronic diseases, and any physical impairments. No students indicated that they had a physical impairment or psychiatric illness (regarding “Other”). The second and third parts comprised questions on exercise and lifestyle characteristics. However, only those items that concerned practicing sports and exercise were included in the analysis. The items were the same in both parts; except that the second part asked about during school years prior to being in college, and the third part asked about currently as a college student. These items asked about the following: regularity of practicing sports per week(NA, 1 = once a week, 2 = twice a week, 3 = thrice a week, 4 = daily); place of practicing the sport (gym, home, walking areas, outdoor playgrounds); type of sports (walking, cardio, swimming, lifting, jumping, diving, boxing, climbing, football, other); reasons for practicing sports (for better health and avoid illnesses, to lose weight, to improve body shape, to enhance muscle strength, to spend free time, to have fun with friends, other); reasons for not practicing sports (time limitation, not interested, have a chronic disease, low income, lack of motivation, feeling tired, unsuitable weather [hot or cold], laziness, no women’s’ gym, other); duration of practicing sports (less than half an hour, half an hour, more than half an hour, more than 1 hour). The third part of the questionnaire also asked for the type of degree they were pursuing. The last question asked if practicing sports affected college attendance (agree, no influence, or neutral). Statistical analysis of the categorical variables; gender, marital status, university,college, academic year, presence of chronic illnesses, type of school, regularity of practicing sport, place of practicing sport, type of sport, reasons for practicing sport, reasons for not practicing sport, period of practicing sport and if physical exercise was affected after attending college. The frequencies and percentages were calculated. For continuous variables; age, the mean and standard deviation were calculated. The paired t-test was used to compare physical exercise prior to college (when attending school) and currently while in college. *P*-values of less than 0.05 were statistically significant. Statistical analyses were performed using SPSS version 21.

## Results

Demographic data on the participants are presented in Table 1. Of the 417 college students who completed the questionnaire,77.2% were women and 22.8% were men. The age of the participants ranged from 16 to 38 years old with a mean age of 21.8 (*SD* = 2.75). More than half (55.9%) were from King Abdulaziz University and healthcare specialties from the different institutions (59.7%).

**Table 1:** Demographic characteristics of the participants (*N* = 417)

Variables	*n*	%
Gender		
Male	95	22.8
Female	322	77.2
Marital status		
Single	373	89.4
Married	44	10.6
Academic year		
Foundation year	21	5.0
2nd year	84	20.1
3rd year	93	22.3
4th year	97	23.3
5th year	50	12.0
Other	72	17.3
Chronic illness		
None	386	92.6
Asthma	9	2.2
Anemia	2	0.5
Hypothyroidism	2	0.5
Diabetes	7	1.7
Muscle and bone diseases	5	1.2
Other	6	1.4
Type of school		
Private school	91	21.8
Public school	317	76.0
Other	9	2.2
Type of degree		
Non degree	1	.2
Bachelor’s	400	95.9
PhD	5	1.2
Diploma	3	.7
Master’s	7	1.7
Flight license	1	.2

No students indicated that they had a physical impairment or psychiatric illness (regarding “Other”).Table 2 presents the results of the frequency of practicing sports, the location, and the reasons for not practicing sports during the school and college years. In college, approximately 39% reported practicing sports or exercising for over half an hour and about 37%did so for up to half an hour. Regarding the reasons for not practicing sports during college, the most common reason was time limitation (18.5%), followed by lack of motivation (16.1%), unsuitable weather (7.2%), and low income (2.6%). Meanwhile, during school years the most common reason was time limitation (29.9%), followed by lack of motivation (12.9%), unsuitable weather (6.2%), and low income (2.2%).

**Table 2:** The frequency, duration, place, and the reasons for not practicing sports and exercise during the school and college years (*N* = 417)

Variables	College years		School years	
	*n*	%	*n*	%
I practice sports regularly				
NA	122	29.3	101	24.2
Once a week	102	24.5	88	21.1
Twice a week	45	10.8	50	12.0
Thrice a week	80	19.2	86	20.6
Daily	68	16.3	92	22.1
I practice sports at^a^				
NA	113	24.9	83	18.1
Home	228	50.3	248	54.0
Gym	105	23.2	115	25.1
Outdoor playgrounds	3	0.7	6	1.3
Walking areas	4	0.9	7	1.5
I do not practice sports due to				
NA	163	39.0	162	38.8
Time limitations	77	18.5	125	29.9
Lack of motivation	67	16.1	54	12.9
Low income	11	2.6	9	2.2
Unsuitable (hot or cold) weather	30	7.2	26	6.2
Not interested in sports	41	9.8	19	4.6
Feeling tired for physical exercise	23	5.5	18	4.3
Other	1	.2	2	.2
Laziness	3	.7	1	.2
There is no women’s gym	1	.2	1	.2
I practice sports for				
NA	99	23.9	65	15.6
Less than half an hour	48	11.5	71	17.0
Half an hour	106	25.4	169	40.5
More than half an hour	161	38.6	108	25.9
More than one hour	3	.6	4	.9

^a^More than one response allowed. *NA* Not applicable

Regarding the type of sports and exercise practiced during college,37.4% practiced one type, 32.4% practiced 2–3 types, and 7.9% practiced 4 or more types of sports and/or exercise. The most common type of physical exercise was walking (61.6%), followed by cardio (28.3%), weightlifting (16.8%), swimming (14.6%), and football (12.2%). Additionally, the most common types during school years were walking (71.7%), cardio (36.5%), weightlifting (26.4%), swimming (17.1%), jumping (11.3%), and football (9.4%).

Table 3 provides the reasons for practicing sports and exercising. While in college, 25.7% had one reason for practicing, 37.7% had 2–3 reasons, and 16% had 4 or more reasons for practicing. The most common reason was to improve body shape (48.0%), followed by losing weight (41.0%), improving health and avoiding illness (39.6%), a way to spend free time (24.5%), building muscle strength (23%), and lastly for fun with friends (13.9%). Meanwhile, during school years, 21.2% had one reason for practicing, 27.8%had 2–3 reasons, and 15.4% had 4 or more reasons for practicing. The most common reason was improving body shape (49.9%), followed by losing weight (42.2%), improving health and avoiding illness (39.8%), building muscle strength (24.5%), spending free time (17.5%), and lastly for fun with friends (7.2%).For the item “my physical exercise was affected after attending college,” 57.1%responded “agree,” 24.7%reported that it had “no influence,” and 18.2% were “neutral.”

**Table 3:** Reasons for practicing sports and exercise during school and college years (*N* = 417)

Variables	College years		School years	
	*n*	%	*n*	%
Reasons for practicing sports^a^				
Building muscle strength	96	23.0	102	24.5
Losing weight	171	41.0	176	42.2
Improving health and avoiding illness	165	39.6	167	40.0
For fun with friends	58	13.9	30	7.2
Spending free time	102	24.5	73	17.5
Improving body shape	200	48.0	208	49.9
No specific reason	0	0.0	53	12.7
Number of reasons for practicing sports				
0	86	20.6	57	13.7
1.00	107	25.7	180	43.2
2.00	85	20.4	59	14.1
3.00	72	17.3	57	13.7
4.00	41	9.8	39	9.4
5.00	26	6.2	25	5.7

^a^More than one response allowed

A paired-samples *t*-test was conducted to compare regularly engaging in sports and exercise before attending college and after entering college. There was a significant difference in the scores for before college (*M* = 3.31, *SD* = 1.47) and since attending college (*M* = 3.05, *SD* = 1.51), *t*(4) = 3.56, *p* < 0.001, indicating less engagement since they have been attending college. Although, when considering the mean values, the difference is minimal, because data were coded for categories, so it’s not easy to conceive the actual difference.

## Discussion

At present, physical exercise is promoted as an important aspect of general health, and the transition from high school to college will pronounce lifestyle changes that can severely challenge a student’s health status [7, 9–11]. Therefore, this study assessed physical exercise practices among university students in Saudi Arabia before beginning and during their college years.

### The regularity of practicing sports and exercise

The results showed that there was a decrease in the regularity of practicing sports or exercising after enrollment in college, which indicates that the decrease found among university students is likely due to the demands of being a student of higher education. This finding is somewhat consistent with the results in a study of Spanish high school students where it was found that there was a decrease in physical activity in the students at the higher grade level (decreased METS walking, increased daily sitting time), although the difference was not statistically significant [17]. Also, the results of a randomized clinical trial showed that there was a significant decrease in exercise by 33% among dental students over their 5-year training program [7]. One of the possible reasons for a decrease in physical activity or exercise might be related to changes in their priorities as they face lifestyle changes, so they became less motivated or able to perform exercise [1, 4, 7]. This brings up an important question as to what curricular initiatives and learning environments influence practicing sports and motivates the regular practice of exercising.

### Reasons for practicing sports and exercise

Understanding the motivations and barriers to participate or not to participate in physical exercise is important because this would help make a balanced exercise program that suits the individual. In our study, the main motivation for practicing sports and exercising during both college and school years was to improve body shape, which was followed by losing weight, followed by improving health and avoiding illness. This contrasts with a study that reported the top reasons that motivated college students to exercise was to remain healthy. The other reasons included gaining the positive feeling that comes from exercise, to join with friends who exercise, and when they feel overweight, in that order [18]. The top reasons may vary between the two studies because our study included students in Saudi Arabia and the prior study was of students in the USA. As such, there may be cultural differences that influence the motivations for exercise.

### Reasons for not practicing sports and exercise

Our study showed that the main reason for not practicing sports and exercising during school years as well as after attending college was due to time limitations. The issue of time limitations is consistent with the findings of a study conducted at Mansoura University in Egypt that found 35.5% of their students were not engaged in physical exercise due to time limitations [15]. Similarly, a study conducted among university students in Kuwait, and another study conducted in Muscat, both reported that not having enough time was one of the main barriers for not practicing sports [16, 19].. Moreover, a study conducted in Spain among 1834 university students concluded that lack of time was the main barrier that prevented them from practicing sports [20]. These findings emphasize that college students have increased demands to achieve academically, and therefore more time is devoted to studying leaving less time for practicing sports and exercise. Thus, available time would be the greatest obstacle that university students would face.

### Types of sports and exercises

There was a significant difference between types of sports engaged in during school and college years. Our findings showed that most of the students before and after attending college preferred walking, but there was a higher preference for walking during school years. Likewise, a study conducted among Chinese students reported that walking was one of the most common forms of exercise preferred by students [21]. The most likely reason walking was the most preferred type is that it is one of the easiest to perform, easy to stick with, safe, has low or no cost associated with it, and does not require any special skills or equipment. Participants in our study preferred cardiovascular fitness during the school years. On the other hand, while in college they preferred football and similar types of sports. In a study conducted at a university in the United States, there was a change in the type of exercise performed by students from their first to the second year of college: stretching exercise performance increased, while there was a decrease in aerobic exercise performance [9]. Thus, it is possible that preferences could change during one’s college years as well. The remaining forms of exercise mentioned were weightlifting, swimming, diving, and jumping, with most being more popular during school years.

### Gender differences

Another finding from the present study was that women were less likely to exercise than men. Several studies have reported similar findings [9, 22, 23]. Moreover, the gender difference was more likely found in Arab women than non-Arab women [23]. Gender differences could be explained by several reasons, as reported by Benjamin and Donnelly [23]: (a) fatigue and tiredness; (b) a lack of social support, and culturally-limited gender role and behavioral expectations for women, where women are expected to stay at home more than men; (c) a lack of sufficient allocation of funding for women’s sports; and (d) a shortage of suitable exercise facilities.

### Place where exercises or sports were for performed

The results revealed that 54% of school students and 50% of college students preferred to exercise at home (as opposed to a gym, playground, or walking areas). No previous studies have assessed this variable (preferred place for exercising among students) and further studies are needed to understand what makes home a preference. It may seem obvious that it is more convenient, but there may be other explanations that could be used to promote exercise in college students. Further research could provide possible explanations.

Our study has several limitations that deserve mentioning. First, the study is limited by its cross-sectional design. As such, we cannot determine that the transition to college was what caused the changes observed. Another limitation is that the majority of the sample were from King Abdulaziz University and health care specialties. Thus, there is a limitation of generalizability. Even though there was a large cross-section of universities and specialties, our results may not be representative of all the students at universities in Saudi Arabia. Further research is needed to investigate and replicate our findings. Additionally, the study is limited by not using a validated questionnaire, rather we used items from other validated questionnaires.

## Conclusion

Physical exercise is important for improving lifelong health and reducing risks of morbidity. The current study clarified the patterns of engaging in sports or exercise among college students. Most students had higher engagement in sports before attending college. More attention should be given to the high rates of non-practice of physical exercise among university students, such as conducting physical exercise awareness campaigns, providing suitable public facilities, and improving the education system.

## Data Availability

The datasets used and analyzed during the current study available from the corresponding author on reasonable request.

## References

[1] Bray SR, Born HA (2004). Transition to university and vigorous physical activity: implications for health and psychological well-being. J Am Coll Heal.

[2] Terenzini PT, Rendon LI, Upcraft ML, Millar SB, Allison KW, Gregg PL (1994). The transition to college: diverse students, diverse stories. Res High Educ.

[3] Conley CS, Kirsch AC, Dickson DA, Bryant FB (2014). Negotiating the transition to college: developmental trajectories and gender differences in psychological functioning, cognitive-affective strategies, and social well-being. Emerg Adulthood.

[4] Meenapriya M, Gayathri R, Vishnu PV (2018). Effect of regular exercises and health benefits among college students. Drug Invent Today.

[5] Keating XD, Guan J, Piñero JC, Bridges DM (2005). A meta-analysis of college students' physical activity behaviors. J Am Coll Heal.

[6] Pinto BM, Marcus BH (1995). A stages of change approach to understanding college students' physical activity. J Am Coll Heal.

[7] Kemmler W, von Stengel S, Kohl M, Bauer J (2016). Impact of exercise changes on body composition during the college years - a five year randomized controlled study. BMC Public Health.

[8] World Health Organization: Global strategy on diet, physical activity and health (DPAS). [https://www.who.int/dietphysicalactivity/pa/en/].

[9] Racette SB, Deusinger SS, Strube MJ, Highstein GR, Deusinger RH (2005). Weight changes, exercise, and dietary patterns during freshman and sophomore years of college. J Am Coll Heal.

[10] Egli T, Bland HW, Melton BF, Czech DR (2011). Influence of age, sex, and race on college students' exercise motivation of physical activity. J Am Coll Heal.

[11] Black DR, Coster DC, Paige SR (2017). Physiological health parameters among college students to promote chronic disease prevention and health promotion. Prev Med Rep.

[12] Booth FW, Roberts CK, Laye MJ (2012). Lack of exercise is a major cause of chronic diseases. Compr Physiol.

[13] Alsubaie AS, Omer EO (2015). Physical activity behavior predictors, reasons and barriers among male adolescents in Riyadh, Saudi Arabia: evidence for obesogenic environment. Int J Health Sci (Qassim University).

[14] Khalaf A, Ekblom Ö, Kowalski J, Berggren V, Westergren A, Al-Hazzaa H (2013). Female university students' physical activity levels and associated factors--a cross-sectional study in southwestern Saudi Arabia. Int J Environ Res Public Health.

[15] El-Gilany AH, Badawi K, El-Khawaga G, Awadalla N (2011). Physical activity profile of students in Mansoura University. Egypt East Mediter Health J.

[16] Musaiger AO, Al-Kandari FI, Al-Mannai M, Al-Faraj AM, Bouriki FA, Shehab FS (2014). Perceived barriers to weight maintenance among university students in Kuwait: the role of gender and obesity. Environ Health Prev Med.

[17] Sánchez-Miguel PA, Leo FM, Amado D, Pulido JJ, Sánchez-Oliva D (2017). Relationships between physical activity levels, self-identity, body dissatisfaction and motivation among Spanish high school students. J Hum Kinet.

[18] Eichorn L, Bruner K, Short T, Abraham SP (2018). Factors that affect exercise habits of college students. J Educ Dev.

[19] Youssef RM, Al Shafie K, Al-Mukhaini M, Al-Balushi H (2013). Physical activity and perceived barriers among high-school students in Muscat, Oman. East Mediterr Health J.

[20] Gómez-López M, Gallegos AG, Extremera AB (2010). Perceived barriers by university students in the practice of physical activities. J Sports Sci Med.

[21] Wu SS, Wang HJ, Li BH, Li SS, Ma J (2010). Association between socioeconomic status and physical activities in Chinese children. Zhonghua Liu Xing Bing XueZaZhi.

[22] Benjamin K, Donnelly TT. Barriers and facilitators influencing the physical activity of Arabic adults: a literature review. Avicenna. 2013;8:2–16.

[23] Kahan D (2015). Adult physical inactivity prevalence in the Muslim world: analysis of 38 countries. Prev Med Rep.

